# Editorial: Women in Pediatric Dentistry Research: Are We There Yet?

**DOI:** 10.3389/fdmed.2023.1172902

**Published:** 2023-03-30

**Authors:** Apoena de Aguiar Ribeiro, Adriana Modesto Vieira

**Affiliations:** ^1^ Division of Diagnostic Sciences, Adams School of Dentistry, University of North Carolina, Chapel Hill, NC, United States; ^2^ Department of Pediatric Dentistry, School of Dental Medicine, University of Pittsburgh, Pittsburgh, PA, United States

**Keywords:** research, pediatric dentistry, women, academia, equity, gender bias

**Editorial on the Research Topic** Women in Pediatric Dentistry Research: Are We There Yet?

Since Lucy Hobbs Taylor, the first woman to earn a degree in dentistry more than 150 years ago, the inclusion of women in dentistry has slowly progressed. Women make up only 30% to 40% of the dental workforce in Oceania, Europe, Africa and Asia (1). Besides the higher number of women in the profession, research shows that women earn less, even after adjusting for specialty and hours worked. Additionally, all else equal, dentists who are women are less likely to own a practice, more likely to be in dental service organizations and more likely to treat Medicaid patients (2).

In Academia, men still comprise the majority of faculty in the US – 61% vs. 37% (3) – and very few women are in academic leadership positions (3–5). The disparity between men and women in senior positions in academic dentistry is even larger around the World and women tend to be positioned in more supportive roles, such as program directors or associate directors at the department level, or assistant and associate deans in health science colleges (1–6). For example, only 16 out of 77 deans in Canadian and American dental schools are women (4–7). Moreover, salary inequity is also observed, where U.S. women earn about 82% of their male counterparts' salaries (8).

In relation to the Pediatric Dentistry as specialty, a recent publication from 2020 investigating the Pediatric Dentistry chairs in the United States and Canada revealed that, despite the similarity in age, women comprised 29.5% and men, 70.5% in the chair positions. Also, women had less leadership training than men, served less in the position and had lower ranking academic titles, spent more time in curriculum and teaching, than in biomedical research (9).

Although women scientists are leading ground-breaking research across the world, we still represent just 33.3% of the scientists (10). Gender gap in publications, grants awarded to women and the general perception in medicine that women are less interested in research have also been reported, in addition to a tendency of placing women in academia to fill administrative and teaching needs, leaving little time to write grants and conduct research (8). As a result, women receive fewer research funding dollars, and they are less likely to be journal editors, principal investigators or members of research boards (7).

In order to change this paradigm, there should be a collective effort worldwide, and gender equality must be promoted and stereotypes defeated. Therefore, we, the Editors, together with Frontiers in Dental Medicine, are extremely proud to offer this Research Topic and promote the work of women researchers across all fields of Pediatric Dentistry. We highlight five publications that comprise this Topic, and expect that this represents a source of inspiration to women in pediatric dentistry research, stimulating bold leadership, research, and solutions that advance women in dental, oral and craniofacial research, and helping promote a systemic change in the near future.

## References

[1] Tiwari T Randall CL Cohen L Holtzmann J Webster-Cyriaque J Ajiboye S Gender inequalities in the dental work-force: global perspectives. Adv Dent Res. (2019) 30:60–8. 10.1177/0022034519877398 31746651 PMC6960321

[2] Versaci MB. HPI: women make up growing percentage of dental workforce. ADANews. (2021). Available at: https://www.ada.org/en/publications/ada-news/2021-archive/march/women-make-up-growing-percentage-of-dental-workforce (Accessed 5, August 2021).

[3] American Dental Education Association. ADEA Snapshot of Dental Education 2020–2021. Available at: C:\Users\apoena\Downloads\ADEA Trends in Dental Education 2020-21.pdf

[4] Li J de Souza R Esfandiari S Feine J. Have women broken the glass ceiling in north American dental leadership? Adv Dent Res. (2019) 30:78–84. 10.1177/0022034519877397 31746652

[5] Ioannidou E D’Souza RN Macdougall MJ. Gender equity in dental academics: gains and unmet challenges. J Dent Res. (2014) 93:5–7. 10.1177/0022034513510178 24144534 PMC3872855

[6] Tandon S Kohli A Bhalla S. Barriers to leadership positions for Indian women in academic dentistry. Intern Dent J. (2007) 57:331–7. 10.1111/j.1875-595X.2007.tb00142.x 17992919

[7] Bompolaki D Pokala SV Koka S. Gender diversity and senior leadership in academic dentistry: female representation at the dean position in the United States. J Dent Educ. (2022) 86(4):401–5. 10.1002/jdd.12816 34741551

[8] Valachovic R. Closing the Gender Gap in Academic Dentistry. Available at: https://adeachartingprogress.wordpress.com/2014/11/14/closing-the-gender-gap-in-academic-dentistry/goo

[9] Townsend JA da Fonseca MA Rodriguez TE LeHew CW. Gender differences in pediatric dentistry chairs in the United States and Canada. J Clin Pediatr Dent. (2020) 44(5):323–31. 10.17796/1053-4625-44.5.6 33181841

[10] https://unesdoc.unesco.org/ark:/48223/pf0000235406.

